# Up-regulation of autophagy by low concentration of salicylic acid delays methyl jasmonate-induced leaf senescence

**DOI:** 10.1038/s41598-020-68484-3

**Published:** 2020-07-10

**Authors:** Runzhu Yin, Xueyan Liu, Jingfang Yu, Yingbin Ji, Jian Liu, Lixin Cheng, Jun Zhou

**Affiliations:** 1 MOE Key Laboratory of Laser Life Science and Guangdong Provincial Key Laboratory of Laser Life Science, College of Biophotonics, South China Normal University, Guangzhou, 510631 China; 2 Department of Critical Care Medicine, Shenzhen People’s Hospital, The Second Clinical Medicine College of Jinan University, Shenzhen, 518020 China; 3 Fujian Provincial Key Laboratory of Plant Functional Biology, College of Life Sciences, Fujian Agriculture and Forestry University, Fuzhou, 350002 China

**Keywords:** Plant hormones, Plant physiology

## Abstract

Crosstalk between salicylic acid (SA) and jasmonic acid (JA) signaling plays an important role in regulation of plant senescence. Our previous work found that SA could delay methyl jasmonate (MeJA)-induced leaf senescence in a concentration-dependent manner. Here, the effect of low concentration of SA (LCSA) application on MeJA-induced leaf senescence was further assessed. High-throughput sequencing (RNA-Seq) results showed that LCSA did not have dominant effects on the genetic regulatory pathways of basal metabolism like nitrogen metabolism, photosynthesis and glycolysis. The ClusterONE was applied to identify discrete gene modules based on protein–protein interaction (PPI) network. Interestingly, an autophagy-related (ATG) module was identified in the differentially expressed genes (DEGs) that exclusively induced by MeJA together with LCSA. RT-qPCR confirmed that the expression of most of the determined ATG genes were upregulated by LCSA. Remarkably, in contrast to wild type (Col-0), LCSA cannot alleviate the leaf yellowing phenotype in autophagy defective mutants (*atg5-1* and *atg7-2*) upon MeJA treatment. Confocal results showed that LCSA increased the number of autophagic bodies accumulated in the vacuole during MeJA-induced leaf senescence. Collectively, our work revealed up-regulation of autophagy by LCSA as a key regulator to alleviate MeJA-induced leaf senescence.

## Introduction

Senescence in green plants is a complex and orderly regulated process that is crucial for transiting from nutrient assimilation to nutrient remobilization^1–5^. During senescence, the most visible characteristic is leaf yellowing, which is the consequence of a succession of changes in cellular physiology including chlorophyll degradation and photosynthetic activity reduction^3,4^. Chloroplast as an early senescence signaling response organelle, its dismantling plays an important role in the major nitrogen source recycling and remobilization^6^. The progression of leaf senescence can be prematurely induced by multiple environmental and endogenous factors, such as temperature, light, humidity and phytohormones^7^. Hormone signaling pathways play roles at all the stages of leaf senescence, including the initiation, progression, and the terminal phases of senescence^7^. Recent progresses show that senescence can be coordinately regulated by several phytohormones like cytokinins, ethylene, abscisic acid, salicylic acid (SA), and jasmonic acid (JA)^8–12^. However, the detailed molecular mechanisms for these phytohormone signals in plant senescence remain poorly understood.

JA has been known as a key plant hormone for promoting senescence, based on the findings that exogenously applied methyl jasmonate (MeJA, methyl ester of JA) leads to a rapid loss of chlorophyll content and accompany with reduction of photochemical efficiency^13,14^. Studies with JA-insensitive mutant *coronatine insensitive 1* (*coi1*) that exhibited defective senescence response to MeJA treatment ^11^, supporting the notion that JA signaling pathway is crucial for leaf senescence. Some other evidences indicate that SA is also involved in plant senescence^12,15^. The concentration of endogenous SA increases to upregulate several senescence-associated genes during leaf senescence^12,16^. However, such genetic regulatory mechanisms are abolished in plants defective in the SA signaling or biosynthetic pathway (*npr1* and *pad4* mutants, and *NahG* transgenic plants)^12^. Crosstalk between MeJA and SA has been broadly documented in plant defense response, which commonly manifests as a reciprocal antagonism pattern^17^. Evidence suggests that antagonistic interactions between SA and MeJA modulate the expression of a senescence-specific transcription factor WRKY53, showing induced by SA, but repressed by MeJA^18^. Overall, mechanisms determining the specificity and coordination between SA and JA still need to be further explored.

Most of phytohormones have both stimulatory and inhibitory effects on the growth and metabolism of higher plants in a dose dependent manner. It seems that SA functions in the same way on the physiological and biochemical processes of plants^14,19^. Low concentrations of SA (hereafter as LCSA) at below 50 micromole (µM) promotes adventitious roots and altered architecture of the root apical meristem, whereas high-concentration SA (greater than 50 µM) inhibits root growth^19^. Interestingly, we previously demonstrated that MeJA-induced leaf senescence could be delayed by LCSA (1–50 μM), but accelerated when the concentration higher than 100 µM^14^. Our other related works have verified such high dose of SA greatly activates NPR1 (nonexpressor of pathogenesis-related genes 1) translocation into nucleus, thereby promoting leaf senescence^15^. Based on the dose dependent effect of SA, Pasternak et al.^19^ proposes that at low levels it acts as a developmental regulator and at high levels it acts as a stress hormone^15^.

Autophagy is associated with plant senescence as defective mutants display early and strong yellowing leaf symptoms^6,20–22^. Autophagy negatively regulates cell death by controlling NPR1-dependent SA signaling during senescence in Arabidopsis^16^. The senescence process always accompanies with the equilibrium between oxidative and antioxidative capacities of the plant, which creates a characteristic oxidative environment resulting in the production of reactive oxygen species (ROS) and more toxic derivatives^23^. Moreover, autophagy is involved in the degradation of oxidized proteins under oxidative stress conditions in Arabidopsis^24^. Actually, there is a complicated interplay between ROS and autophagy, i.e., ROS can induce autophagy while autophagy be able to reduce ROS production^25^. Our previous studies showed that LCSA application delays senescence by enhancing the activities of antioxidant enzymes and restricting reactive oxygen species (ROS) accumulation in MeJA-treated leaves^14^. However, it is still unclear whether autophagy pathway is implicated in the LCSA-alleviated leaf senescence.

In this study, the relationships between SA and MeJA in plant senescence were further investigated. By applying transcriptome and interaction network analysis, we identified an autophagy-related (ATG) gene module in the differentially expressed genes (DEGs) that induced by MeJA together with LCSA. In contrast to wild type (Col-0), LCSA cannot alleviate the leaf yellowing phenotype in autophagy defective mutants upon MeJA treatment. Further results revealed that LCSA increased the number of autophagic bodies during MeJA-induced leaf senescence. Collectively, our work provides new insight that up-regulation of autophagy by LCSA act as a critical regulating process to alleviate MeJA-induced leaf senescence.

## Materials and methods

### Plant materials and chemical treatments

Arabidopsis plants of wild-type (WT, Col-0), *atg5-1* (SAIL_129_B07), *atg7-2* (GK-655B06) and eYFP-ATG8e^26^ were grown in a greenhouse at 22 °C with 16 h light photoperiod (120 μmol quanta^−2^ m^−2^). Phytohormones treatment was performed as described by Ji et al.^14^. Briefly, the 3rd and 4th rosette leaves from four weeks of plants were detached and incubated in 3 mM MES buffer (pH 5.8) containing 50 μM methyl jasmonate (MeJA) and/or 10 μM salicylic acid (SA). MeJA was prepared from a 50 mM stock solution in ethanol. Solutions without MeJA were supplemented with equal amounts of ethanol. Concanamycin A (ConcA) was prepared as a 1 mM stock solution in DMSO and used at final concentration 1 μM.

### Photochemical efficiency and chlorophyll content measurements

The photochemical efficiency was measured with an Imaging-PAM Chlorophyll Fluorometer (PAM-MINI, Walz, Germany) followed the procedure described previously^27^. After dark-adapted for 1 h, parameters Fo (minimum fluorescence with PSII reaction centers fully open) and Fm (maximum fluorescence after dark adaptation) were acquired with a 0.8-s saturating pulse (4,000 μmol photons m^-2^ s^-1^). The value of Fv/Fm was calculated by the formulas (Fm-Fo)/Fm. Chlorophyll was extracted by immersion in 90% ethanol at 65 °C for 2 h. The absorbance at 664 nm and 647 nm were determined with a Lambda 35 UV/VIS Spectrometer (Perkin-Elmer)^28^. The concentration per fresh weight of leaf tissue was calculated according to the formula: micromoles of chlorophyll per milliliter per gram fresh weight = 7.93(A664) + 19.53(A647). The percentages of Fv/Fm and chlorophyll content are calculated relative to the initial levels of samples before treatment (time zero).

### RNA-Seq analysis

Detached 3rd and 4th rosette leaves from 4-week old plants were immersed in 3 mM MES buffer (pH 5.8) containing 10 μM SA, 50 μM MeJA, and MeJA together with SA for 24 h. Total RNA for RNA-Seq was extracted from leaves using a plant RNA kit (Magen, China). Purified RNA was analyzed either using a ND-1000 Nanodrop (Thermo Fisher, USA), or by agarose gel electrophoresis to determine the RNA quantity. Those RNA samples with no smear seen on agarose gels, a 260/280 ratio above 2.0, and RNA integrity number greater than 8.0 were used. For RNA-Seq analysis, three replication samples for each treatment were mixed into one, and total RNA samples were then sent to RiboBio Co., Ltd (Guangzhou, China) for sequencing. The NEBNext Poly(A) mRNA Magnetic Isolation Module (NEB, USA) was used for mRNA purification. The Ultra II RNA Library Prep Kit for Illumina was used for RNA library construction. The libraries were sequenced as 50-bp single end reads using Illumina Hiseq2500 according to the manufacturer's instructions.

### Differential expression analysis

Raw read count of each gene was generated using HTSeq with union-count mode^29^. After normalization by Reads Per Kilobase per Million mapped reads (rpkm), normalized read count table was used for determining differentially expressed genes (DEGs)^30–32^, which were defined as those with twofold changes. Fold change was calculated using log2 (normalized read count + 1). An R package *clusterprofiler* was used to perform the functional category analysis to detect the significantly enriched Gene Ontology (GO) terms^33,34^. Significantly enriched GO terms were selected by a threshold of p ≤ 0.05. Protein–protein interaction (PPI) data was obtained from the STRING database (v.10, https://string-db.org) ^35^. To construct a high-confidence network, only the PPIs with confidence scores larger than 0.7 were considered in this work. ClusterONE was adopted for the identification of protein clusters or functional modules using default parameters as described previously^36,37^. The protein modules including five or more than five members and having connection density over 0.5 are defined as modules.

### RT-qPCR

Total RNA was isolated using a Super RNA Kit (Promega, Shanghai, China) and genomic DNA was removed using DNase I. 1 μg of RNA was used to make cDNA with the Reverse Transcription System (Promega, Shanghai, China). For qPCR 10 μL of 2X qPCR-S Mastermix (Sangon, Shanghai, China) and 1 μL of cDNA (100 ng/μL) for a total of 20 μL was used in each well. Real-time PCR was done on a Real-Time System (BioRad) at 95 °C for 2 min, and 45 cycles of 95 °C for 15 s, 55 °C for 30 s, and 72 °C for 30 s followed by a melting curve analysis. For each sample 3 biological reps were used and repeated 3 times for technical replication. qPCR was analyzed using the ΔΔCt method. Primers for qPCR were showed in Table S2. Statistical significance was determined using Duncan’s multiple range test.

### Confocal microscopy

Detached 3rd and 4th rosette leaves were immersed in 3 mM MES buffer (pH 5.8) containing 50 μM MeJA and/or 10 μM SA for 24 h. ConcA (1 μM) was added at 6 h before confocal imaging. Confocal images were captured with 63x (numerical aperture [NA], 1.4) objective using an LSM 880 microscope (Zeiss). For quantification of autophagic puncta, randomly selected 15 to 20 images for each three independent experiments were quantified with ImageJ. All images were collected with the same settings determined prior to the experiment to yield nonsaturating conditions.

## Results

### LCSA delays MeJA-induced leaf senescence

**Figure 1:**
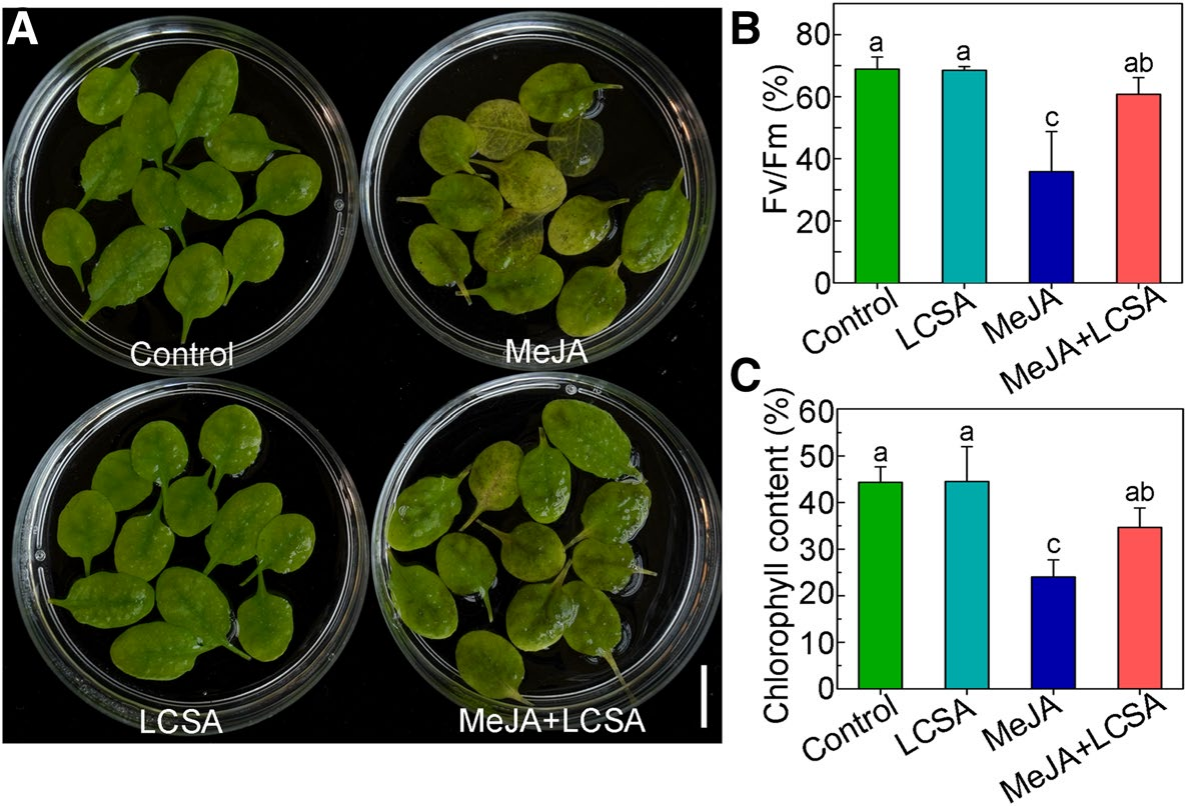
LCSA alleviates MeJA-induced leaf senescence. (**A**) Phenotypes of detached leaves under LCSA and/or MeJA treatments. The 3rd and 4th rosette leaves were incubated in 3 mM MES buffer (pH 5.8) containing LCSA (10 μM) or MeJA (50 μM) alone or in combination (MeJA + LCSA) under continuous light for 5 d. Scale bar, 20 mm. (**B** and **C**) Measurement of the maximum quantum efficiency of photosystem II (PSII) photochemistry (Fv/Fm) (**B**) and total chlorophyll content (**C**) after LCSA and/or MeJA treatments. The percentages of Fv/Fm and chlorophyll content are relative to the initial levels at time zero. Data were the mean ± SE of three independent experiments. Different letters indicate statistically significant differences between each treatment (Duncan’s multiple range test, *p* < 0.05).

Our previous works indicated that SA delays MeJA-induced leaf senescence at low concentrations (1–50 μM)^14^. On this basis, 10 μM SA, the most effective concentration according to Ji et al.^14^, was selected as low working solution to further investigate the effect of LCSA on leaf senescence. As shown in Fig. 1, in contrast to control, LCSA did not appear to have a discernible effect on leaf senescence. Leaves incubated with MeJA (50 μM) were greatly turned yellow after 5 days treatment. However, when MeJA worked together with LCSA (MeJA + LCSA), the leaf yellowing phenotype was alleviated (Fig. 1A). Consistently, the photochemical efficiency Fv/Fm and loss of chlorophyll content in the leaves that combined treatment with LCSA and MeJA were less severe relative to that of the leaves treated with MeJA alone (Fig. 1B,C). These physiological and biochemical data are consistent with our previous finding that LCSA provide protection against senescence caused by MeJA.

### Expression patterns of genes in LCSA-induced delayed leaf senescence

**Figure 2:**
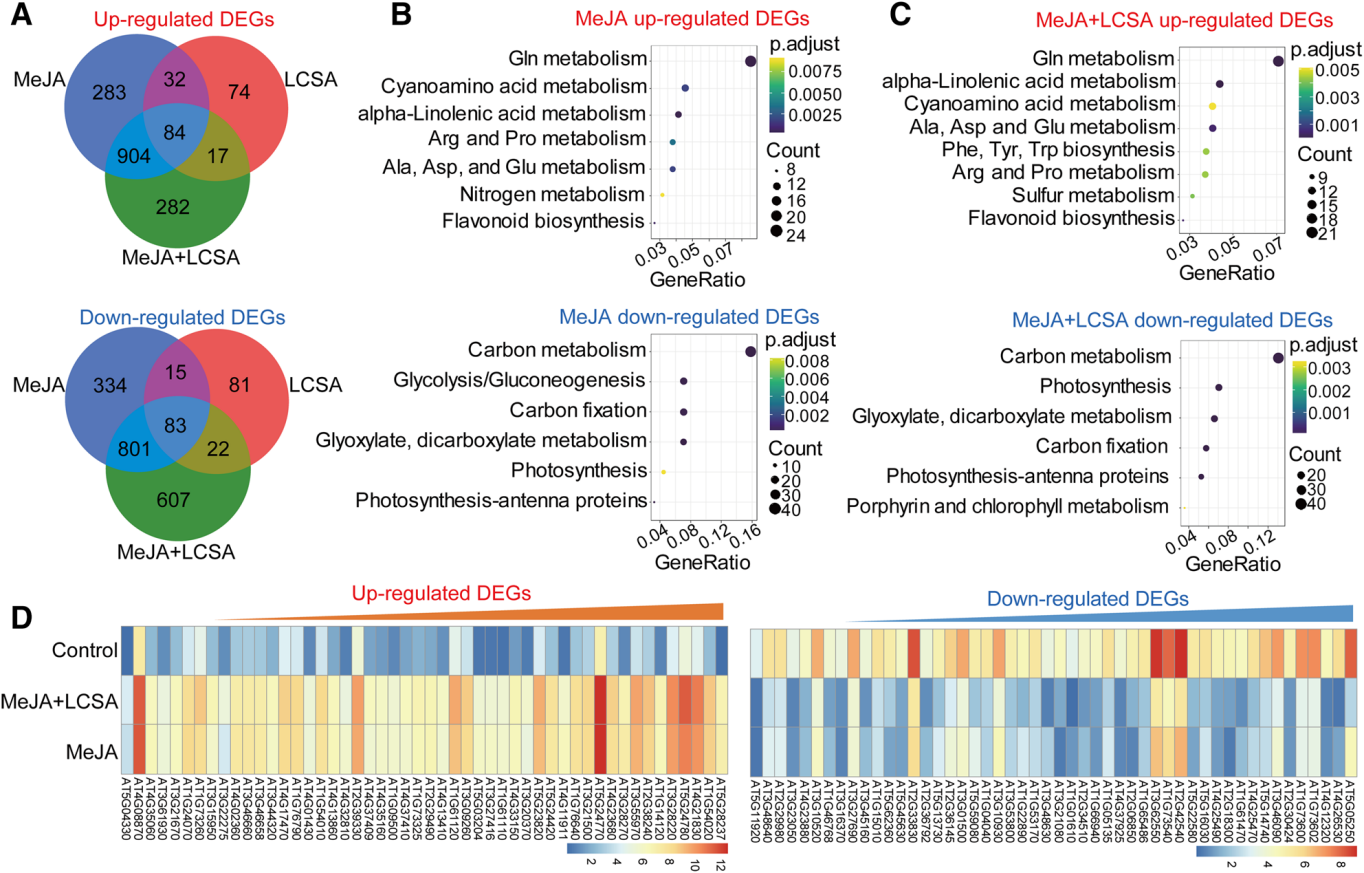
RNA-Seq analyses of differentially expressed genes (DEGs) in samples treated with LCSA, MeJA and MeJA + LCSA. (**A**) Venn diagram showing the overlap of DEGs between LCSA, MeJA and MeJA + LCSA -treated samples. (**B** and **C**) The pathway enrichment analysis of up or down -regulated DEGs induced by MeJA alone (**B**) or MeJA + LCSA (**C**). (**D**) The heatmap showing expression of top 50 up-regulated and down-regulated DEGs between MeJA and MeJA + LCSA treatment group.

To investigate the genome-wide effect of LCSA on MeJA-induced gene expression changes, we performed RNA-sequencing experiments. Since gene transcription regulation occurs prior to visible phenotype, leaves treatment with phytohormones at 1 d were selected according to our previous study^14^. Totally, 408, 2,536 and 2,800 genes displayed at least twofold changes in the expression level of LCSA, MeJA, and MeJA + LCSA -treated leaves, respectively, relative to control leaves (Fig. 2A). Of these, the number of differentially expressed genes (DEGs) of LCSA alone were greatly less than that in MeJA or MeJA + LCSA treatment group, in consistent with the inconspicuous phenotype between SA and control leaves (Fig. 1). Therefore, our study is mainly concentrated on the differential expression of genes between MeJA and MeJA + LCSA.

To interpret the up-regulated and down-regulated DEGs resulting from the MeJA and MeJA + LCSA treatment, functional enrichment of Gene Ontology (GO) terms was performed using the hypergeometric test (*P* value < 0.05). The analysis of biological process GO terms illustrated that most of the induced DEGs related to amino acid (Glutathione, Cyanamino acid, arginine, proline, alanine, aspartate and glutamate) metabolism, nitrogen metabolism, and flavonoid biosynthesis, whereas, the repressed DEGs mainly related to carbon metabolism, photosynthesis, and glycolysis (Fig. 2B). These features of nitrogen and carbohydrate metabolism are consistent with the senescing phenotype of leaves. In contrast to MeJA alone, unexpectedly, LCSA together with MeJA treatment did not make much differences on the enriched biological processes (Fig. 2C). The heatmap illustrated the top 50 up-regulated and down-regulated DEGs, which also revealed an extremely similar expression pattern between the DEGs of MeJA and MeJA + LCSA treatment (Fig. 2D). These results indicated that LCSA does not appear to have dominant effects on the genetic regulatory network of basal metabolism like nitrogen metabolism, photosynthesis, and glycolysis.

### Network analysis identifies autophagy-related gene module

**Figure 3:**
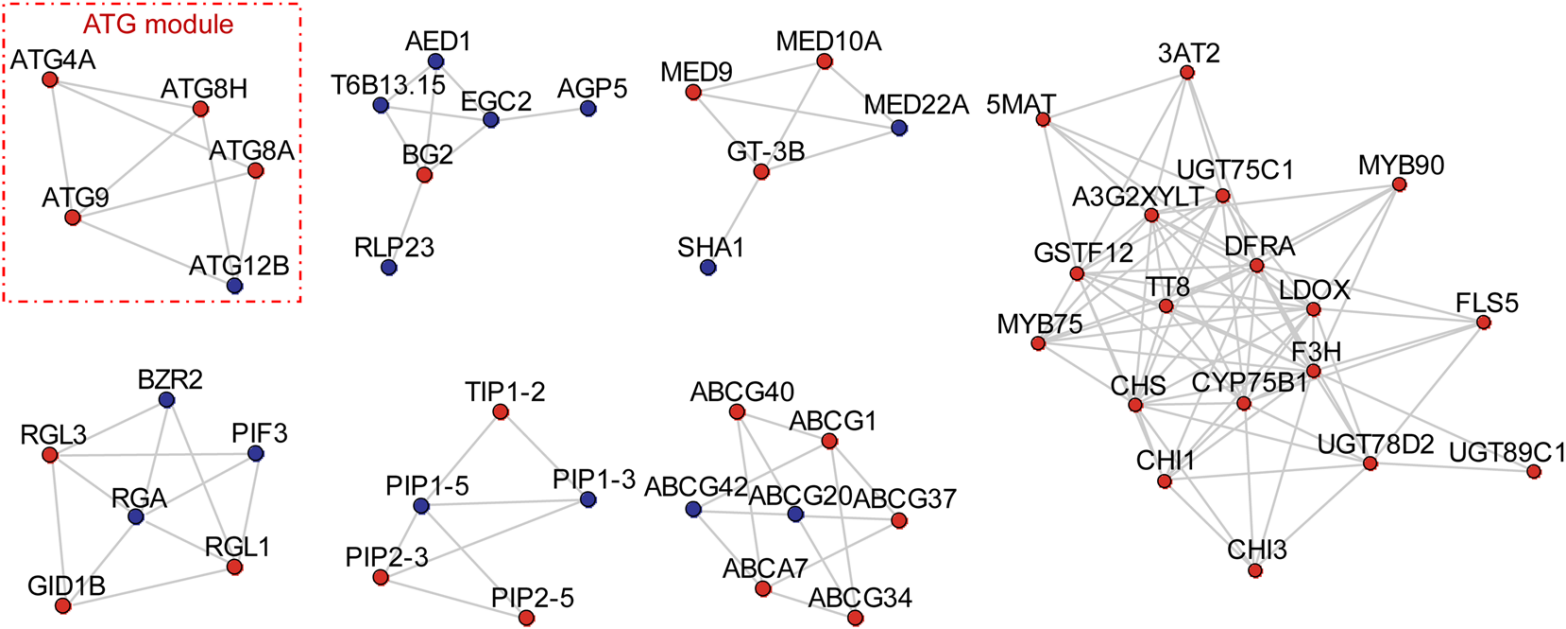
Network analysis identifies distinct signal modules in the DEGs exclusively induced by MeJA + LCSA treatment. Interconnected clusters enriched among the 889 genes and their interactions with neighboring genes. The autophagy specific module was drawn in a red dotted line. Genes are colored in red if they are induced and in blue if they are repressed.

Since the enrichment analysis only provided undifferentiated biological processes about basal metabolism, network analysis was conducted using DEGs that induced by MeJA and MeJA + LCSA, respectively. The protein–protein interactions (PPI) were collected from the STRING database, and only the PPIs with confidence scores higher than 0.7 were selected, resulting in a high confidence network with 719,964 interactions and 17,372 proteins. ClusterONE was used to identify functional protein modules, which were defined by the protein clusters including five or more than five members and having connection density over 0.5^37^. According to such screening specifications, we identified 15 gene modules in MeJA treatment group and 16 gene modules in MeJA + LCSA group, respectively (Figure S1 and S2). Of these, six gene modules were specially detected in the MeJA treatment group (Figure S3). Interestingly, MeJA together with SA exclusively induced seven gene modules, covering genes involved in autophagy-related pathway, phytohormone response, ATP-binding cassette transporters, aquaporins, and flavonoid biosynthesis (Fig. 3). In this context, autophagy is an essential intracellular degradation system that plays important roles in nutrient remobilization during leaf senescence^6^. We found that the transcript abundance for ATG proteins (ATG4, ATG8, ATG9, and ATG12) was differentially sensitive to the MeJA + LCSA treatment. From the enriched biological processes and molecular functions, we observed that these ATGs are the core components that contribute to autophagosome mature and biogenesis. Collectively, these results suggest a framework in which MeJA together with LCSA regulates the abundance of specific gene network, such as the autophagy process.

**Figure 4:**
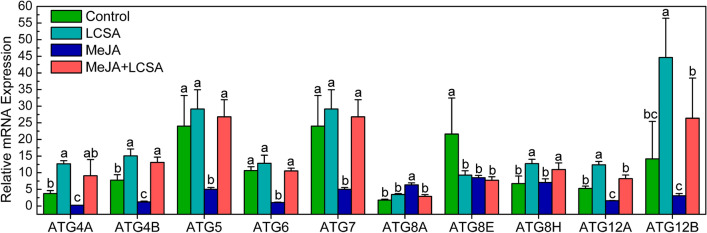
RT-qPCR confirmation of differentially expressed genes that involved in regulation of autophagy. The relative mRNA expression levels were calculated using the ΔΔCt method. The values of each ATG gene were relative to the initial levels at time zero of treatment. Data were the mean ± SE of three independent experiments. Different letters in each gene indicate statistically significant differences between the treatments (Duncan’s multiple range test, *p* < 0.05).

We next investigated whether the autophagy pathway was involved in LCSA-delayed leaf senescence. Ten ATG genes (ATG4A, ATG4B, ATG5, ATG6, ATG7, ATG8A, ATG8E, ATG8H, ATG12A, and ATG12B) that implemented in autophagosome formation were examined by RT-qPCR (Fig. 4). In contrast to MeJA alone, most of these determined ATG genes, except for ATG8A and ATG8E, were up-regulated by the combined treatment group (MeJA + LCSA). The differential gene expression of ATG8 isoforms is possible due to they have different expression pattern in distinct tissues^20^. Interestingly, it should be mentioned that MeJA together with LCSA did not stimulate a much more increase in gene expression compared with control, especially LCSA treatment (Fig. 4). These results indicate that restoration of ATG genes expression is closely related to LCSA-delayed leaf senescence.

### SA-delayed leaf senescence is dependent on a functional autophagy pathway

**Figure 5:**
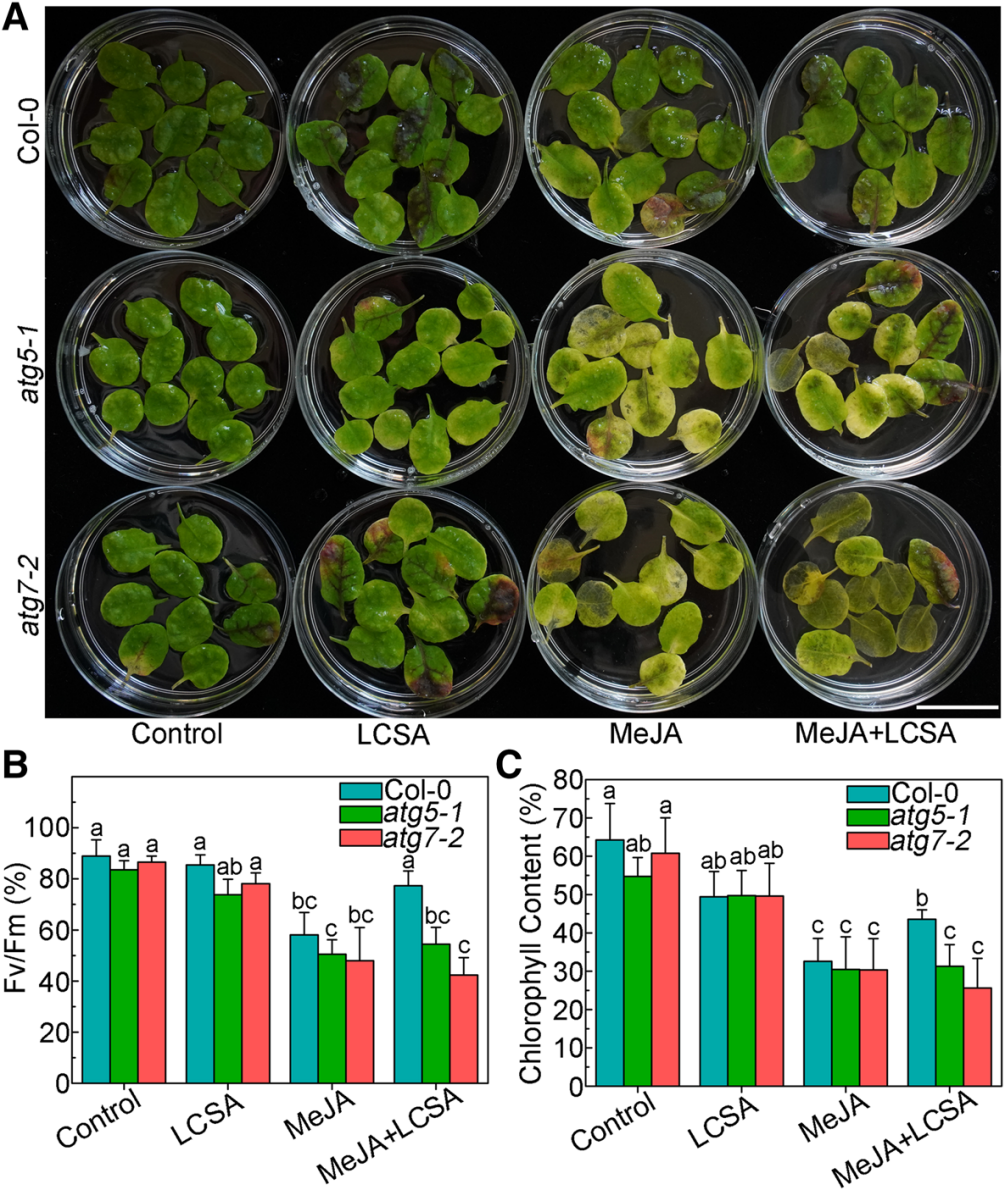
Defective in autophagy restrains the effect of LCSA on the senescence symptoms. (**A**) Leaf phenotypes of LCSA-alleviated senescence in Col-0 and autophagy defective mutants (*atg5-1* and *atg7-2*). Detached leaves from four-week-old Col-0, *atg5-1*, and *atg7-2* plants were transferred to MES buffer (pH 5.8) containing LCSA (10 μM) or MeJA (50 μM) or both MeJA and LCSA (MeJA + LCSA) under continuous light. Photographs were taken after 5 days of treatment. Scale bar, 20 mm. (**B** and **C**) Relative Fv/Fm (**B**) and chlorophyll levels (**C**) in the leaves of the Col-0, *atg5-1*, and *atg7-2* described in (**A**). Data were the mean ± SE of three independent experiments. Different letters indicate statistically significant differences between the treatments (Duncan’s multiple range test, *p* < 0.05).

To further resolve whether autophagy pathway was crucial for LCSA-delayed leaf senescence, two autophagy defective mutants (*atg5-1* and *atg7-2*), that involved in ATG8 lipidation during phagophore elongation^38^, were analyzed upon LCSA and/or MeJA treatment. In contrast to wild type (Col-0), leaves from *atg5-1* and *atg7-2* mutants were showed much more yellowing after incubated with MeJA for 5 days (Fig. 5A). As expect, the leaf yellowing phenotype was not alleviated when MeJA worked together with LCSA (Fig. 5A). Consistently, the photochemical efficiency Fv/Fm in the *atg5-1* and *atg7-2* mutant leaves treated with MeJA + LCSA was not restored relative to that of the leaves treated with MeJA (Fig. 5B). Similarly, none of the two mutants had recovered relative chlorophyll content as the Col-0 after combined treatment with MeJA and LCSA (Fig. 5C). These genetic results clearly illustrated that the protection against MeJA-induced senescence by LCSA is dependent on a functional autophagy pathway.

### SA increases autophagy activity upon MeJA-induced leaf senescence

**Figure 6:**
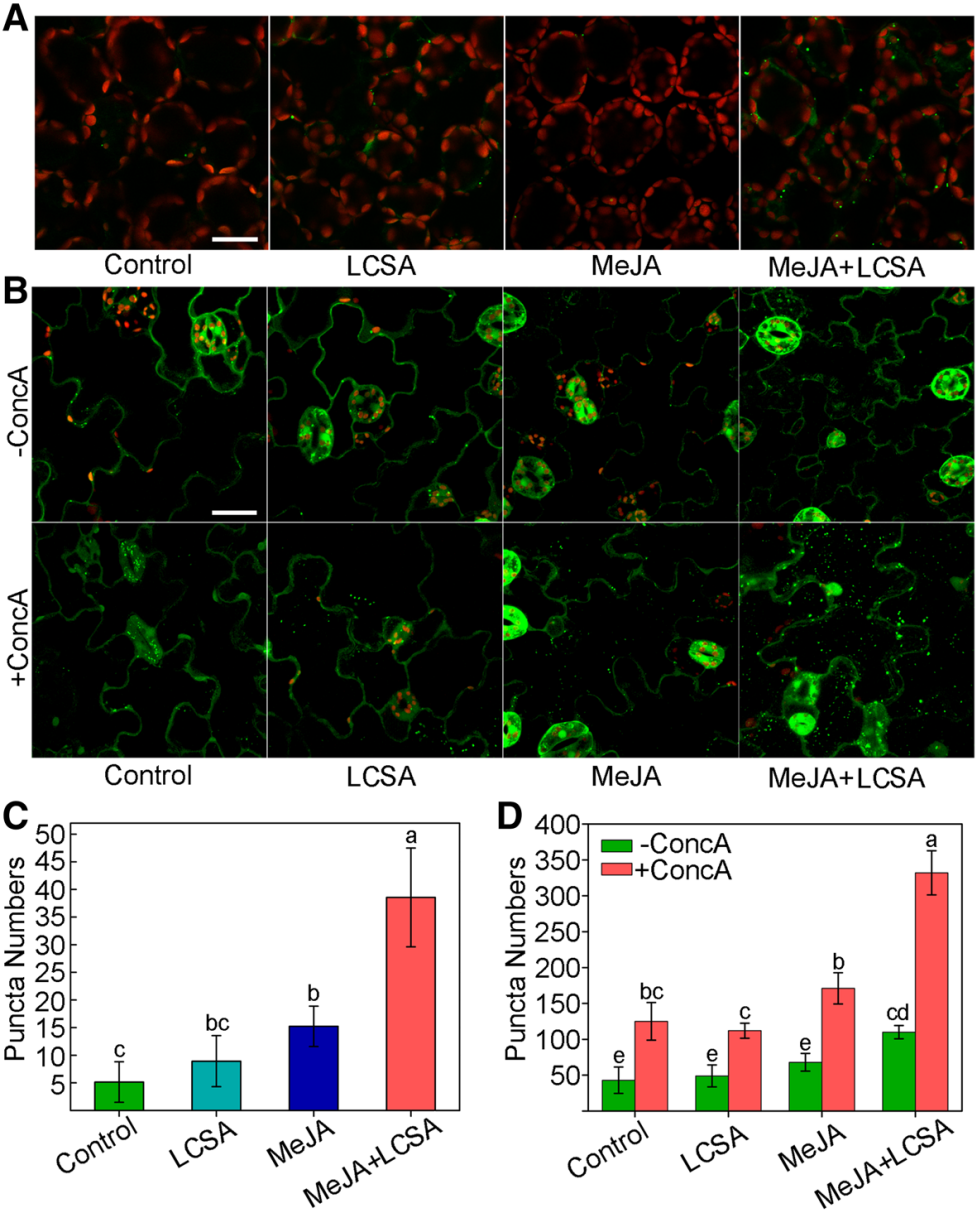
LCSA enhances the formation of autophagosomes upon MeJA-induced leaf senescence. (**A**) Microscopic analyses of autophagosome-related structures in the mesophyll cells of eYFP-ATG8e plant under LCSA or MeJA or MeJA + LCSA treatment. (**B**) Examination of autophagic bodies accumulated in the vacuoles. ConcA, concanamycin A. Bar = 20 μm. (**C** and **D**) Statistical analysis of the puncta numbers displayed in (**A**) and (**B**), respectively. The numbers of puncta were calculated per 0.01 mm^2^ from at least 15 pictures. This experiment was repeated in triplicate with similar results. Different letters indicate statistically significant differences between the treatments (Duncan’s multiple range test, *p* < 0.05).

Since autophagy pathway was verified involved in LCSA-delayed leaf senescence, we next further determined the detailed autophagy activity. Wild-type Arabidopsis plants expressing the eYFP-ATG8e fusion protein were subjected to LCSA and/or MeJA treatment, and the effects of LCSA on autophagy activity were analyzed by confocal microscopy of the YFP fluorescence. In control and LCSA treatment conditions, we observed a few fluorescent punctate structures that were identified previously as ATG8-tagged autophagosomes (or autophagic bodies)^26^. Incubation of MeJA alone induced a slightly increase in accumulation of autophagic bodies (Fig. 6A,B). However, when the detached leaves were subjected to combined treatment with MeJA and LCSA, there was a greatly increase in the fluorescent vesicles (Fig. 6A,B). The statistical results showed that the number of autophagic bodies was more than twofold higher in MeJA + LCSA group than that of treatment with MeJA alone (Fig. 6C,D). Taken together, our observations collectively suggest that LCSA activates the autophagy activity to delay MeJA-induced leaf senescence.

## Discussion

As the final stage of leaf development, leaf senescence is a complex process that involves thousands of genes and multiple layers of regulation. Mechanisms governing the specificity regulation of phytohormones and output gene expression are therefore of great interest. The primary objective of the work is to further explore the crosstalk between SA and JA signaling in regulating plant leaf senescence. We have concentrated on examining the mechanisms likely to underpin changes in the transcriptome in response to LCSA and/or MeJA. Specifically, an autophagy module was identified from the DEGs that exclusively induced by MeJA together with SA (Fig. 3). Further results demonstrate that the upregulation of autophagy by LCSA serves important function in alleviating MeJA-induced leaf senescence (Figs. 5 and 6).

Previously, we found that SA delays MeJA-induced leaf senescence in a concentration dependent manner^14^. The dosage-dependent effect of SA also has been reported in plant root meristem regulation. SA at low levels (below 50 µM) promotes adventitious roots and alters architecture of the root apical meristem, whereas high-concentration SA (> 50 µM) inhibits root growth^19^. Such discrepancies are probably due to SA acts as a developmental regulator at low levels, but acts as a stress hormone at high levels^19^. Interestingly, RNA-Seq results showed that the number of DEGs in LCSA alone treatment was less than MeJA or LCSA and MeJA combined treatment group (Fig. 2A), which consistent with LCSA itself did not have a discernible effect on senescence, showing the inconspicuous phenotype between LCSA and control leaves (Fig. 1). Moreover, in contrast to MeJA alone, LCSA together with MeJA treatment did not make much differences on the biological process of GO terms (Fig. 2C). These results indicated that LCSA at low level is more likely function as a signaling regulator, which does not have a marked impact on the basal metabolism at least at the genetic regulatory level.

Autophagy promotes cell survival by adapting cells to stress conditions both in plants and mammals. Recent reverse-genetic studies have revealed that autophagy is closely associated with plant senescence, and autophagy defective mutants like *atg2*, *atg5* and *atg7* all showed early yellowing leaf symptoms^16,39^. SA is one of the most promising phytohormones that contribute to the induction of autophagy under stress. There is a complicate relationship between the SA-induced autophagy and autophagy-exerted SA signaling. Mutation of ATG5 caused a high level of SA accumulation in Arabidopsis^16^. On the other side, autophagy could negatively regulate cell death by controlling NPR1-dependent SA signaling during senescence^16^. Here, the ClusterONE was applied to identify discrete gene modules based on PPI network. We identified several modules including autophagy-related network in DEGs that exclusively induced by MeJA together with LCSA (Fig. 3). The protection against MeJA-induced senescence by LCSA was abolished in autophagy defective mutants *atg5-1* and *atg7-2* (Fig. 5). These data strongly suggest an important role for autophagy in LCSA-alleviated leaf senescence. Interestingly, in contrast to the similar transcript level of ATG8e, a huge number of puncta was induced by MeJA + LCSA treatment (Figs. 4 and 6). The autophagy markers eYFP-ATG8e was expressed under the *ubiquitin-10* gene promoter^26^. There was a relative few puncta at the control condition, even it has strong fluorescent signal. Therefore, the inconsistency between transcript levels of ATG8e and numbers of puncta with ATG8e might owing to some post-transcriptional modifications on the ATG components that enhanced autophagy after treatment with MeJA + LCSA.

Notably, unlike the greatly increase of autophagic bodies induced by MeJA + LCSA, autophagosomes under LCSA alone treatment were not statistically significant when compared with control (Fig. 6). Nevertheless, it is worth pointing out that SA at 100 µM, a high-concentration that could promote leaf senescence based on our previous study^15^, greatly induced autophagic structures formation (Figure S4). Moreover, MeJA itself slightly increased the number of ATG8e puncta (Fig. 6). During leaf senescence, the autophagic activity is increased to clean up the toxic proteins and damaged organelles^6^. It is a relatively rational result that a slightly increased autophagic activity to cope with MeJA-induced leaf senescence. In this context, we speculate that the function of LCSA is similar to the priming activators but with a slight difference, because it does not need time memory to result in a robust autophagy response. Actually, the priming induced by some plant activators (e.g. *β*-aminobutyric acid, and thiamine) are dependent on SA signaling^40–42^. It would be interesting to test the priming effect of LCSA on leaf senescence in future research.

In summary, this study further investigated the interactions between SA and MeJA in plant senescence. Several modules including an autophagy-related (ATG) cluster were identified by analyzing the transcriptome data and protein interaction networks. Further results showed that LCSA could upregulate autophagy to alleviate MeJA-induced leaf senescence. This was confirmed by founding that LCSA cannot alleviate the leaf yellowing phenotype in autophagy defective mutants upon MeJA treatment. Collectively, our work reveals LCSA tend to function as a signaling regulator to upregulate autophagy pathway, which serves as an important cellular mechanism responsible for alleviation of MeJA-induced leaf senescence.

## Supplementary information

Supplementary Information.

## Data Availability

RNA-seq data were deposited in the Sequence Read Archive (SRA) database https://www.ncbi.nlm.nih.gov/sra with accession no. PRJNA578602.
