# Supplementary material for: Up-regulation of autophagy by low concentration of salicylic acid delays methyl jasmonate-induced leaf senescence
Source: Sci Rep. 2020 Jul 10;10:11472. doi: 10.1038/s41598-020-68484-3 (PMC7351724; doi:10.1038/s41598-020-68484-3)
Supplement: Supplementary file 1 — Supplementary Information. [file 41598_2020_68484_MOESM1_ESM.pdf]

Up-regulation of autophagy by low concentration of salicylic acid delays methyl jasmonate-induced leaf senescence

Runzhu Yin[1#], Xueyan Liu[2#], Jingfang Yu[1], Yingbin Ji[1,3], Jian Liu[4], Lixin Cheng[2*], Jun Zhou[1*]

1 MOE Key Laboratory of Laser Life Science & Guangdong Provincial Key Laboratory of Laser Life Science, College of Biophotonics, South China Normal University, Guangzhou 510631, China

2 Department of Critical Care Medicine, Shenzhen People's Hospital, The Second Clinical Medicine College of Jinan University, Shenzhen 518020, China

3 Luoyang Tmaxtree Biotechnology Co., Ltd, Luoyang 471023, China

4 Fujian Provincial Key Laboratory of Plant Functional Biology, College of Life Sciences, Fujian Agriculture and Forestry University, Fuzhou 350002, China

SUPPLEMENTARY MATERIALS

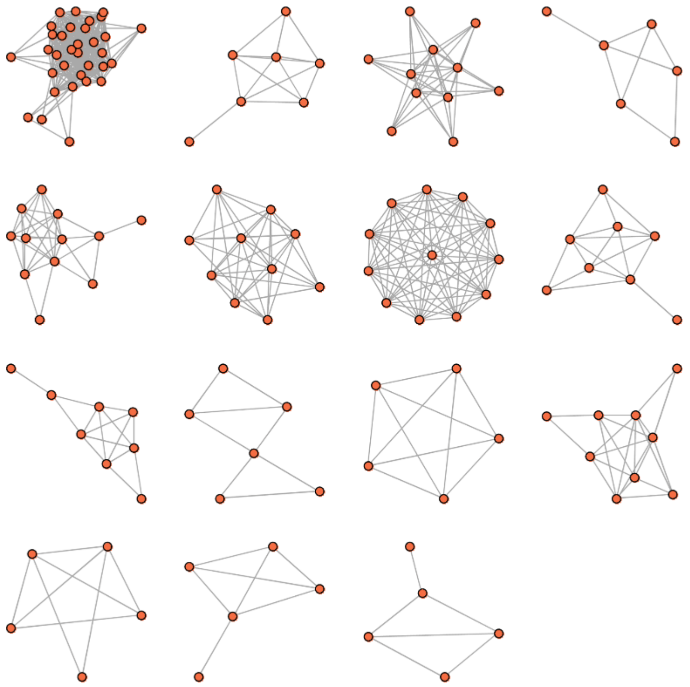

Figure S1. Network analysis identifies gene modules in the DEGs induced by MeJA. Protein modules were identified using ClusterONE, a cluster screen method considering the overlapping neighbor extension. The protein modules including five or more than five members and having connection density over 0.5 are defined as modules.

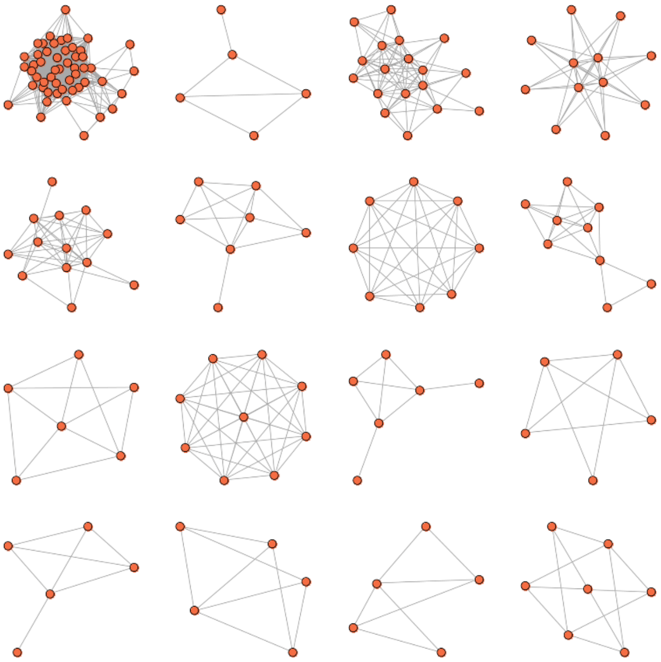

Figure S2. Network analysis identifies gene modules in the DEGs induced by MeJA together with LCSA. Protein modules were identified using ClusterONE. The protein modules including at least five members and having connection density over 0.5 are defined as modules.

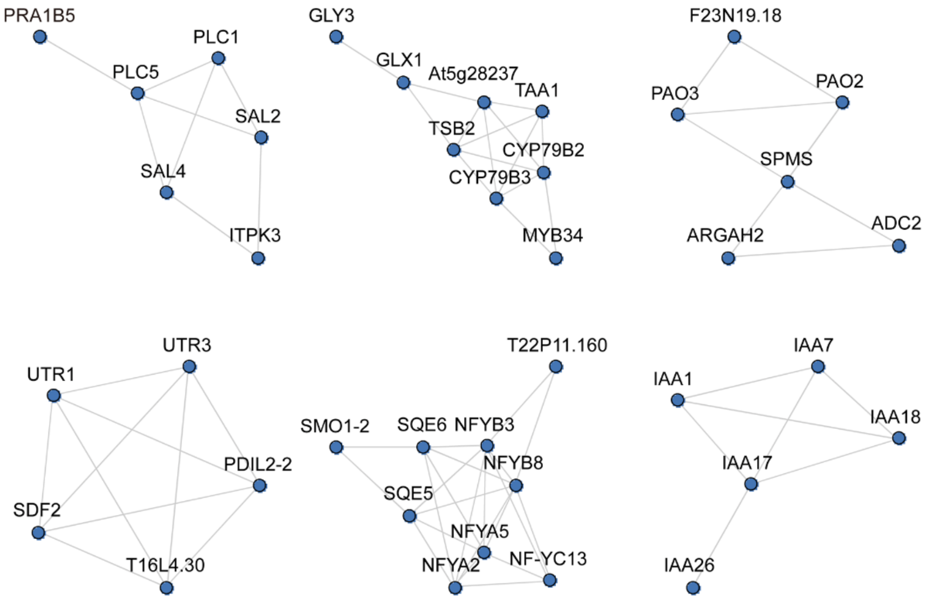

Figure S3. Gene modules exclusively induced by MeJA. After removal of the same modules in MeJA+LCSA treatment group, six gene modules specially induced by MeJA were obtained.

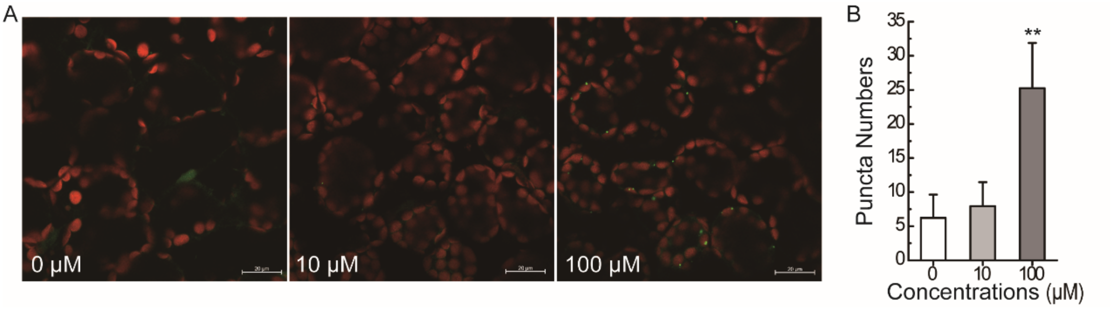

Figure S4. Effect of different concentrations of SA on autophagic puncta induction. (A) Microscopic analyses of autophagic structures in the eYFP-ATG8e plant under 0, 10 and 100 M SA treatment. Bar, 20 μm. (B) Statistical analysis of the puncta numbers displayed in (A). The number of puncta was calculated per 0.01 mm^2 from at least 15 pictures. Asterisks indicate a significant difference according to Student's t-test, **P<0.01.

Table S1. Genes list for enriched modules in the DEGs induced by MeJA and MeJA together with LCSA.

Modules	Enriched genes in each module
MeJA_1	AT1G64200 ATCG00120 ATCG00480 ATCG00140 ATCG00190 ATCG00740 ATCG00170 ATCG00340 AT3G48850 ATCG00660 ATCG01070 ATCG01110 ATCG00420 ATCG01050 ATCG00490 AT5G24120 ATCG00280 ATCG00510 AT5G13730 ATCG01010 ATCG01100 ATMG01320 ATCG00570 ATCG00550 ATCG00710 ATCG00210 AT5G13490 ATCG00560 ATCG00040 ATCG00590 ATCG00760 ATCG00300 ATCG00700
MeJA_2	AT4G36990 AT3G24520 AT3G51910 AT3G53230 AT5G37670 AT2G20560 AT1G08650
MeJA_3	AT3G50740 AT4G36220 AT5G04330 AT3G19450 AT4G37990 AT1G71695 AT5G64120 AT3G49120 AT4G21960 AT4G08770 AT5G06720
MeJA_4	AT5G58690 AT5G58670 AT5G01640 AT4G08170 AT5G64000 AT5G09290
MeJA_5	AT1G30135 AT1G74950 AT5G13220 AT3G17860 AT1G70700 AT1G17380 AT3G43440 AT2G27690 AT2G34600 AT5G05600 AT1G44350 AT5G63450
MeJA_6	AT3G29320 AT5G64860 AT5G04360 AT3G20440 AT2G36390 AT5G51820 AT1G76130 AT4G25000 AT2G39930 AT4G00490
MeJA_7	AT3G48360 AT3G26230 AT3G26300 AT4G37400 AT1G13100 AT4G37310 AT5G57220 AT4G37320 AT4G37430 AT5G36220 AT3G53280 AT4G37410
MeJA_8	AT1G19670 AT5G43860 AT4G13250 AT5G13800 AT4G22920 AT3G44880 AT3G10520 AT4G11910
MeJA_9	AT1G70560 AT1G11840 AT5G28237 AT4G27070 AT1G53580 AT4G39950 AT2G22330 AT5G60890
MeJA_10	AT5G53120 AT1G62810 AT2G43020 AT3G59050 AT4G08870 AT4G34710
MeJA_11	AT1G04980 AT1G14360 AT2G02810 AT2G25110 AT4G29520
MeJA_12	AT5G02570 AT5G43250 AT4G22756 AT5G24160 AT5G24150 AT4G14540 AT2G37060 AT3G05690 AT1G54160
MeJA_13	AT1G02470 AT2G43018 AT3G59052 AT5G10690 AT2G06005
MeJA_14	AT1G51950 AT3G23050 AT1G04250 AT4G14560 AT3G16500
MeJA_15	AT1G52400 AT4G19230 AT3G16470 AT1G52410 AT5G24780
MeJA+LCSA_1	ATCG00470 ATCG00130 AT2G25610 AT1G64200 ATCG00120 ATCG00480 ATCG00150 ATCG00140 ATCG00770 ATCG00740 ATCG00170 ATCG00670 ATCG00020 ATCG00630 ATCG01060 AT3G48850 ATCG00650 ATCG00660 ATCG00640 ATCG01070 ATCG00430 ATCG00420 ATCG01080 ATCG01050 ATCG00490 ATCG00540 ATCG00080 ATCG00580 ATCG00070 ATCG00510 AT1G16780 AT3G27240 AT3G53920 ATCG01100 AT2G07689

	ATCG00440 ATCG01040 ATCG01020 ATCG00570 ATCG00550 ATCG00360 ATCG00520 ATCG00710 ATCG00210 AT5G13490 ATCG00220 ATCG00560 ATCG00040 ATCG00590 ATCG00760 ATCG00530 ATCG00700
MeJA+LCSA_2	AT1G52400 AT4G19230 AT3G16470 AT1G52410 AT5G24780
MeJA+LCSA_3	AT1G03495 AT4G14090 AT5G42800 AT5G54060 AT5G17220 AT3G29590 AT1G06000 AT3G51240 AT5G17050 AT5G13930 AT4G09820 AT4G22880 AT5G07990 AT5G63600 AT1G56650 AT1G66390 AT5G05270 AT3G55120
MeJA+LCSA_4	AT1G14540 AT5G40150 AT3G50740 AT4G36220 AT5G04330 AT4G37990 AT1G14550 AT1G71695 AT5G64120 AT3G49120 AT4G21960 AT5G05340
MeJA+LCSA_5	AT1G01260 AT1G30135 AT1G74950 AT1G72450 AT5G13220 AT3G17860 AT1G70700 AT1G17380 AT2G27690 AT2G34600 AT5G05600 AT1G44350 AT5G63450
MeJA+LCSA_6	AT1G19670 AT4G13250 AT5G13800 AT4G22920 AT3G44880 AT3G10520 AT4G11910
MeJA+LCSA_7	AT3G29320 AT5G64860 AT2G36390 AT5G03650 AT1G76130 AT4G25000 AT2G39930 AT4G00490
MeJA+LCSA_8	AT4G36990 AT3G24520 AT3G51910 AT1G77570 AT3G53230 AT1G57870 AT1G08650 AT1G73570 AT1G65040
MeJA+LCSA_9	AT1G09530 AT1G19350 AT2G01570 AT5G17490 AT1G66350 AT3G63010
MeJA+LCSA_10	AT3G26230 AT2G30750 AT4G37310 AT3G26220 AT5G57220 AT4G37320 AT4G37430 AT5G36220 AT4G37410
MeJA+LCSA_11	AT1G35230 AT2G18660 AT5G10760 AT3G57260 AT2G14610 AT2G32680
MeJA+LCSA_12	AT1G02470 AT2G43018 AT3G59052 AT5G10690 AT1G51402
MeJA+LCSA_13	AT1G16430 AT5G41910 AT2G38250 AT1G55080 AT5G63780
MeJA+LCSA_14	AT4G21980 AT3G06420 AT2G44140 AT2G31260 AT3G13970
MeJA+LCSA_15	AT1G01620 AT4G23400 AT3G26520 AT2G37180 AT3G54820
MeJA+LCSA_16	AT2G39350 AT3G47780 AT3G53510 AT1G15520 AT4G15233 AT2G36380 AT3G53480

Table S2. Primers used for RT-qPCR. F, Forward; R, Reverse.

Primer name	Sequence (5'--->3')
Actin2-F	GGCAAGTCATCACGATTGG
Actin2-R	CAGCTTCCATTCCCACAAAC
ATG4A-F	GGCTGCATTGCAACTAGATTT
ATG4A-R	GAATCATGCAACCCCAGTTC
ATG4B-F	CTTTCACGTTCCCTCAAAGC
ATG4B-R	TTGCAATGGTAAGACGATGTG
ATG5-F	GACAGCAAGAATTCCTGTTCG
ATG5-R	GGAACTCAACAGGGCGATTA
ATG6-F	GCCATGACACTGTATTTGATG
ATG6-R	TGGTCCAACTCTCTTGCTTG
ATG7-F	GTACCGCTTGCTCTGAAACC
ATG7-R	GTCTTCCCAGTCGAGGTTGA
ATG8A-F	CAATTTGTATACGTGGTTCGT
ATG8A-R	AGCAACGGTAAGAGATCCAA
ATG8E-F	GGAAGCATCTTTAAGATGGACA
ATG8E-R	CTCAGCCTTTTCCACAATCA
ATG8H-F	CCAAAGCTCTCTTTGTTTTCG
ATG8H-R	AAGAACCCGTCTTCTTCCTTG
ATG12A-F	CGAGCTCTGTTCGGAAAGTT
ATG12A-R	CAACGAATCAGAATGAAGCTG
ATG12B-F	CCGAATCTCCGAATTCTGTT
ATG12B-R	GCAAACTTGTCACTCCCTGA
